# Application of random forest based approaches to surface-enhanced Raman scattering data

**DOI:** 10.1038/s41598-020-62338-8

**Published:** 2020-03-25

**Authors:** Stephan Seifert

**Affiliations:** 1 Kiel University, University Hospital Schleswig-Holstein, Institute of Medical Informatics and Statistics, Kiel, 24105 Germany; 2 Present Address: University of Hamburg, Hamburg School of Food Science, Institute of Food Chemistry, Hamburg, 20146 Germany

**Keywords:** Machine learning, Analytical chemistry, Raman spectroscopy, Biophotonics

## Abstract

Surface-enhanced Raman scattering (SERS) is a valuable analytical technique for the analysis of biological samples. However, due to the nature of SERS it is often challenging to exploit the generated data to obtain the desired information when no reporter or label molecules are used. Here, the suitability of random forest based approaches is evaluated using SERS data generated by a simulation framework that is also presented. More specifically, it is demonstrated that important SERS signals can be identified, the relevance of predefined spectral groups can be evaluated, and the relations of different SERS signals can be analyzed. It is shown that for the selection of important SERS signals Boruta and surrogate minimal depth (SMD) and for the analysis of spectral groups the competing method Learner of Functional Enrichment (LeFE) should be applied. In general, this investigation demonstrates that the combination of random forest approaches and SERS data is very promising for sophisticated analysis of complex biological samples.

## Introduction

Surface-enhanced Raman scattering (SERS) is an analytical approach that is capable to study small structures in biological materials^1^ and that is even able to detect single molecules^2,3^. Because SERS can also be applied as *in vitro* analytical tool^4^, e.g. to analyze living cells^5,6^ it has the potential to become the next generation sensor technology to monitor cells and tissues^7^. Hence, SERS has been widely applied, for example to study blood^8^, bacteria^9^, viruses^10^, cancer^11^, and to develop a pH sensor in living cells^12^. SERS analyzes the local environment of nanoparticles that are utilized as nanoprobes which can result in very diverse SERS spectra in environments with many different biomolecules. Hence, one of the main challenges of biological SERS applications is the question of how to obtain reliable and interpretable results. One possible solution is the application of SERS labels, nanoparticles that are combined with functionalized reporter molecules for specific binding and, hence, to obtain more reproducible and specific SERS spectra^7^. However, in this case, usually only the signals of the reporter molecules are detected.

Another approach that can also be applied to the spectra of reporter molecules is the analysis of the SERS data with multivariate statistical methods. This combination has for example been used to classify bacteria^13^ and for cell imaging^14^. In this context, usually unsupervised methods like principal component analysis (PCA) and hierarchical cluster analysis (HCA) are applied. However, in label-free SERS experiments it has been shown that variation due to the nature of SERS can hamper analysis with PCA and HCA when biological samples containing multiple molecules are analyzed^15^. This can be circumvented when supervised methods like artificial neural networks (ANN) are applied. Although ANNs have been utilized, e.g. to quantify caffeine^16^, food dye^17^, and metabolite gradients^18^ and to discriminate DNA^19^, they have not been applied to SERS data to a great extend. One reason for this rare use and a major drawback of many machine learning methods including ANN is that the resulting prediction models are black boxes. Random forest (RF)^20^, however, is a machine learning approach that consists of multiple decision trees, which makes it relatively easy to peek into the black box. As a result multiple random forest based approaches have been developed for the identification of important predictor variables mainly for the purpose of analyzing high dimensional omics data like gene expression data^21–26^. The objective of this study is to apply different RF based approaches to SERS data to evaluate the possibilities that they provide to extract useful information from this challenging and unique data type. More precisely the RF based methods Vita^23^, Boruta^24^, minimal depth (MD)^25^ and surrogate minimal depth (SMD)^26^ are applied to select important variables and Learner of Functional Enrichment (LeFE)^27^ and prediction error (PE)^28^ to evaluate predefined groups. In addition, SMD is also utilized to analyze variable relations. To comprehensively evaluate the validity and power of the different methods, the true properties of the data needs to be known which is why a framework for the simulation of SERS data was established for this study.

## Methods

### SERS data simulation

For the simulation a compromise between two opposite characteristics had to be found: A relatively simple structure should be used that can easily be interpreted and shows clear findings. On the other hand surface-enhanced Raman scattering (SERS), especially when it is applied to complex, biological samples, generates quite complex data. This results from the nature of the SERS experiment where the current environment of nanoparticles is probed. Thus, in the presence of various biomolecules the observed SERS signals can be very different and the simulated spectra should be very diverse.

As an easily understandable basis for the simulation, so-called single spectra were generated and used as a straightforward proxy for SERS information that originate from one analyte or from various analytes that co-occur in the SERS spectra. In order to obtain realistic and diverse SERS spectra these single spectra were subsequently combined with random proportions. Consequently the simulation can be subdivided into two parts: The simulation of twelve single spectra and the combination of these single spectra to obtain SERS data sets.

In order to generate single spectra in the wavenumber region from 300 to 1700 cm^−1^ with a resolution of 1 cm^−1^, for each spectrum 1401 spectral data points were obtained from a normal distribution with a mean of 200 and a standard deviation of 50 corresponding to baseline and noise of the spectra, respectively. Subsequently, a randomly selected number of bands between three and ten consisting of a random band maximum position and a randomly selected band width between 5 and 20 cm^−1^ were drawn for each of the spectra. For each band a gaussian curve with a maximum value between 1000 and 10000 and a standard deviation of the respective band width was simulated and added to the spectrum at the respective positions, so that the maximum of the gaussian curve corresponds to the band maximum position.

In the second step two different groups of spectra were built combining two to five of the twelve single spectra for each SERS spectrum. The first 500 spectra contained single spectrum 1 as characteristic spectrum with characteristic bands and the second 500 spectra single spectrum 2. The other ten single spectra appear in both groups and, hence, are group unspecific SERS signals that determine the variable background of the group characteristic bands. Only a fraction *f* of the spectra of the respective group contains the characteristic single spectrum and this fraction was altered utilizing the values 0, 0.05, 0.2, 0.5, and 0.8. The data set with *f* = 0 represents a null scenario with SERS spectra for both groups that do not contain any characteristic bands. For the characteristic spectra in the SERS data sets the proportion *w* of the respective characteristic single spectrum was randomly chosen from values between 0.1 and 0.8. The remaining proportion of the SERS spectrum (0.9 to 0.2 in total) was defined by one to four of the additional single spectra. The SERS spectra were obtained by adding up the contributing single spectra multiplied by the respective proportions. For each value of *f* 50 replicates, each containing 1000 SERS spectra (500 for each group), were simulated. Hence, the total data set for each value of *f* contained 50000 spectra (50  ⋅  1000 spectra) and, in total, 250000 spectra were obtained. Finally, all of the SERS spectra were vector-normalized, meaning that the intensity of each spectral variable was divided by the sum of the intensities of the respective spectrum.

A schematic overview of the simulation process is given in Fig. S1 and an R markdown script to generate the data is provided as supplementary material.

### Random forest (RF)

Random forest is a machine learning approach that utilizes a large number of individual decision trees that are obtained by different subsets (bootstrap samples) of the training data^20^. In the tree building process the optimal split for each node is identified from a set of randomly chosen candidate variables. Besides their application to predict the outcome in classification and regression analyses, RF can also be applied to select important variables and groups, and to enable a deeper understanding of variable relations.

### Software and analyses

The software R (version 3.5.2) and the R packages Pomona in version 1.0.0 (for Boruta and Vita, https://github.com/silkeszy/Pomona), SurrogateMinimalDepth in version 0.1.9 (for MD and SMD, https://github.com/StephanSeifert/SurrogateMinimalDepth), and PathwayGuidedRF in version 0.2.3 (for PE and LeFE, https://github.com/szymczak-lab/PathwayGuidedRF) all using the ranger package^29^ for random forest generation were utilized. The parameters applied for ranger and the RF based approaches are summarized in Table 1. For each analysis, classification mode to differentiate the two groups with different characteristic spectra was used.

**Table 1:** Parameters used for the random forest based approaches.

Approach	Parameter	Description	Value
RF	ntree	number of trees	1000 (LeFE and PE)
			10000 (Vita, Boruta, SMD, MD)
	mtry	number of candidate predictor variables	228 (number of variables^(3∕4)^)
	nodesize	number of spectra in terminal node	1
Vita	p.t	threshold for p-values	0
Boruta	pValue	confidence level	0.01
	maxRuns	maximal number of importance source runs	100
SMD	s	predefined number of surrogate splits	70 (number of variables ∗0.05)
	t	user defined factor to calculate threshold	1
PE	no.perm	number of permutations	100
LeFE	sample.factor	multiple of number of variables selected	1
		outside of variable group (“pathway”)	
	sample.runs	number of repetitions of comparison	75

### Selection of important variables

Various methods have been developed to identify important variables based on random forests. Most of them use the permutation importance that is calculated as the difference of the prediction performance of a variable before and after the permutation of the variable’s values. Approaches differ in the way they distinguish important variables with a large permutation importance from unimportant variables with a small permutation importance.

The main idea of the Boruta algorithm^24^ is to compare the importance of the real variables to those of so-called shadow variables that are added to the data set. Shadow variables are obtained by permuting a copy of the real variables across observations to destroy the relationship with the outcome. Subsequently a RF is trained on the extended data set, the permutation importance values are collected and for each real variable the respective importance is compared to the maximum value of all shadow variables using a statistical test. Afterwards variables with significantly larger or smaller importance values are labeled as important or unimportant, respectively. The unimportant variables are removed and the whole process is replicated until all variables are labeled or a predefined number of runs has been performed.

The Vita algorithm^23^ is based on the idea that most variables in omics data sets are unimportant and can be used to estimate importance values of null variables. Here, the observed non-positive permutation importance scores that are obviously obtained from unimportant variables are used to estimate a distribution that is symmetric around zero. Based on this empirical distribution, p-values for all variables are calculated and important variables can be separated from unimportant variables based on a p-value threshold.

A different approach to select important variables independently of the permutation importance is based on the tree structures of the random forest. Minimal depth (MD) variable importance is calculated by the average layer of first appearance of a variable in the decision trees^25^. Here, variables with MD values below a threshold are labeled as important. The threshold is calculated based on the average MD of non-relevant variables in a hypothetical setting where the outcome is independent of all predictor variables.

In order to include variable relations in tree structure based variable importance measures, surrogate minimal depth (SMD) has been developed that incorporates surrogate variables into the concept of MD^26^. Surrogate variables have originally been proposed to replace missing values in the data^30^ and are obtained in addition to the primary split, in order to be able to replace this split as good as possible. In SMD the importance measure of first appearance is not only applied to the primary split variable but also to the surrogate split variables. The threshold to identify important variables with low SMD values is calculated based on the average SMD of non-relevant variables in a hypothetical setting where the outcome and all of the variables are independent of each other.

The performance of Boruta, Vita, MD and SMD was compared using different evaluation criteria. In addition to the selection frequencies of each variable in comparison to the characteristic spectra, the sensitivity and the false positive rate of variable selection was calculated. The false positive rate was obtained by the fraction of the falsely identified variables and the total number of variables that are not part of any characteristic band. The sensitivity was determined by the division of the correctly identified variables and the total number of variables of the characteristic bands. Furthermore, the sensitivity of band identification was determined by the division of the number of bands from which any variable was selected by the total number of characteristic bands.

### Selection of related variables

The concept of surrogate variables in RF decision trees can not only be exploited to optimize the selection of important variables but also to analyze variable relations^26^. For this purpose the parameter adjusted agreement that is used in the process of finding the best surrogate variables can be utilized and mean adjusted agreement values for potentially related variables are obtained. These mean adjusted agreement values are compared to a threshold that is calculated from the mean adjusted agreement of a hypothetical setting with unrelated variables to identify related variables with higher values. In order to demonstrate the selection of related variables one replicate of the data set with *f* = 0.8 was analyzed and mean adjusted agreement values for the band maximum at 1323 cm^−1^ were reported.

A detailed analysis of the variable relations was conducted between the 13 maxima band variables of the characteristic single spectra and the results are summarized in a relation matrix. Each cell of the matrix gives the proportion of replicates in which the relation was detected, i.e. the mean adjusted agreement exceeded the threshold. For visualization the R package ComplexHeatmap^31^ was used and the ordering of the variables was determined by k-means clustering that is performed separately for the rows and columns utilizing Euclidean distance. For the null scenario the previously described characteristic band analysis was also performed for the nine band maxima of the single spectra 8 and 12.

### Evaluation of predefined spectral groups

In order to include group membership information in the RF analysis, pathway-guided RF approaches have been developed. Here pathways, meaning variable groups, are analyzed instead of individual variables and the approaches Learner of Functional Enrichment (LeFE)^27^ and prediction error (PE)^28^ were applied.

LeFE trains RFs on a variable data set that consists of the group variables and a multiple number of randomly selected variables that are not part of the group. Subsequently a statistical test is applied to decide if the importance scores of the group are significantly larger than the scores from outside the group. Because the number of total variables in SERS data is considerably smaller than in omics data the same number of variables inside and outside of the group (and consequentially a sample factor of 1 in Table 1) was used.

PE trains a RF for each group and compares the prediction error of out-of-bag samples (samples that are not part of the respective bootstrap sample) to an empirical null distribution that is obtained by permuting the outcome values. For this comparison a statistical test is applied, as well^32^.

As variable groups the variables of the bands of the twelve single spectra were utilized and the selection frequency of each group over all replicates was determined.

## Results and discussion

### Simulated SERS data

In order to evaluate the results of the comparison of different random forest (RF) based approaches accurately the underlying mechanisms and effects have to be known which is why simulated data were used (see Fig. S1 for a schematic overview of the data simulation). In the simulation process twelve single spectra (see Fig. S2a–l) as a proxy for co-occuring SERS signals were generated and combined to obtain realistic SERS spectra. To create a classification setting for the analysis two different groups of spectra were built. Each group exclusively contained one of the first two single spectra called characteristic spectra, while both groups contained the other ten single spectra called background spectra.

**Figure 1:**
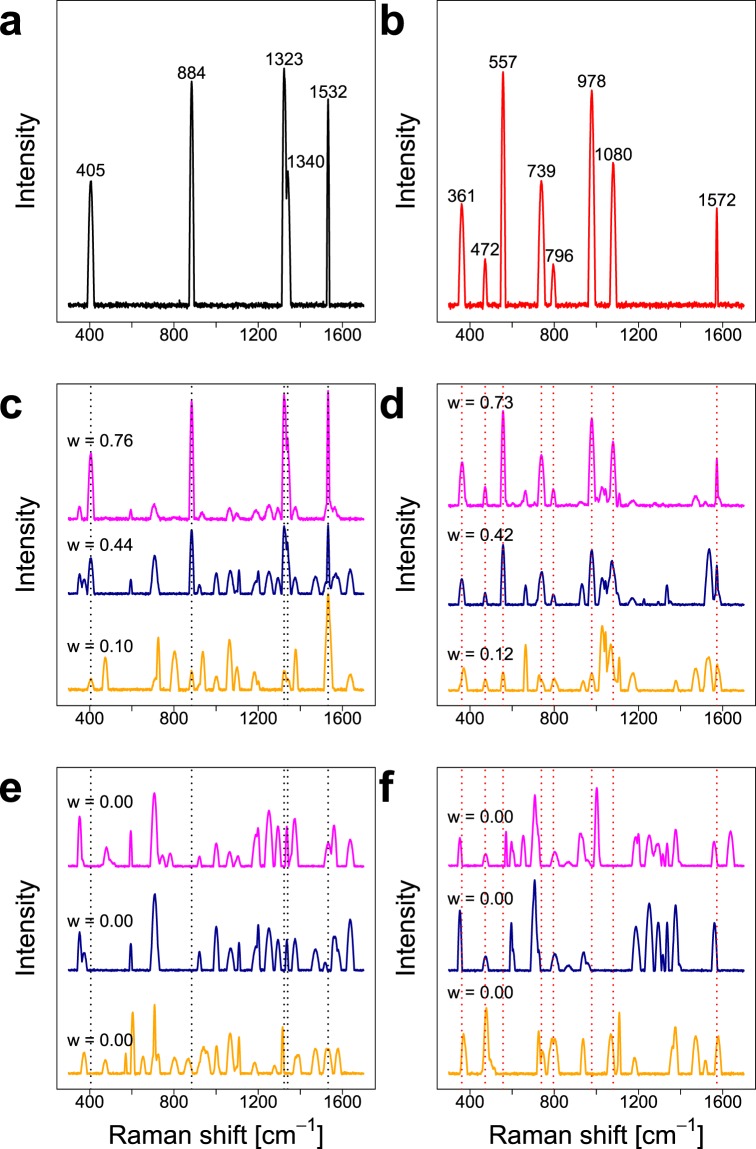
Characteristic single spectra for the two groups (**a,b**) and examples for SERS spectra of the two groups containing (**c,d**) and not containing (**e,f**) the respective characteristic spectrum. The parameter *w* displays the proportion of the respective characteristic single spectrum in the SERS spectrum. This figure was generated using the software R^33^ (version 3.5.2).

Figure 1a,b show the characteristic single spectra of the two groups and the simulated bands show different peak positions, width and intensities. In Fig. 1c,d representative SERS spectra containing the characteristic spectra in high (0.76 and 0.73), medium (0.44 and 0.42) and low (0.10 and 0.12) proportions *w* are depicted exemplarily and Fig. 1e,f show spectra of the two groups that only contain background single spectra. It can be concluded that SERS spectra were obtained that show realistic characteristics, e.g. diverse signals that are partially characteristic for the respective group. Because the two different groups were generated utilizing one respective single spectrum exclusively, the analysis results can simply be compared to the characteristic single spectra to assess the performance of the random forest based approaches.

### Selection of important variables

First, various RF based methods to select important variables were evaluated. Vita and Boruta clearly performed best in a recent comparison study of permutation based approaches on high-dimensional data sets^34^ and, hence, they were also applied here. In addition, the decision tree structure based minimal depth (MD) and surrogate minimal depth (SMD) were utilized.

Figures S3–S7 show the selection frequencies of each individual variable for the different SERS data sets with different fractions *f* of the characteristic spectra. Since the characteristic spectra are defined as the SERS signals that are different between the two groups, the bands of these spectra are the true important variables that should be identified by the variable selection approaches. The figures show that all of the approaches frequently select variables of the characteristic bands. However, a comparison of the different methods based on this visualization is not straightforward, which is why sensitivity and false positive rate were calculated. Because the actual aim of the selection is the identification of important bands and not the identification of all band variables the sensitivity was calculated for the identification of bands. This means that a characteristic band is correctly identified when any of the bands variables is selected.

**Figure 2:**
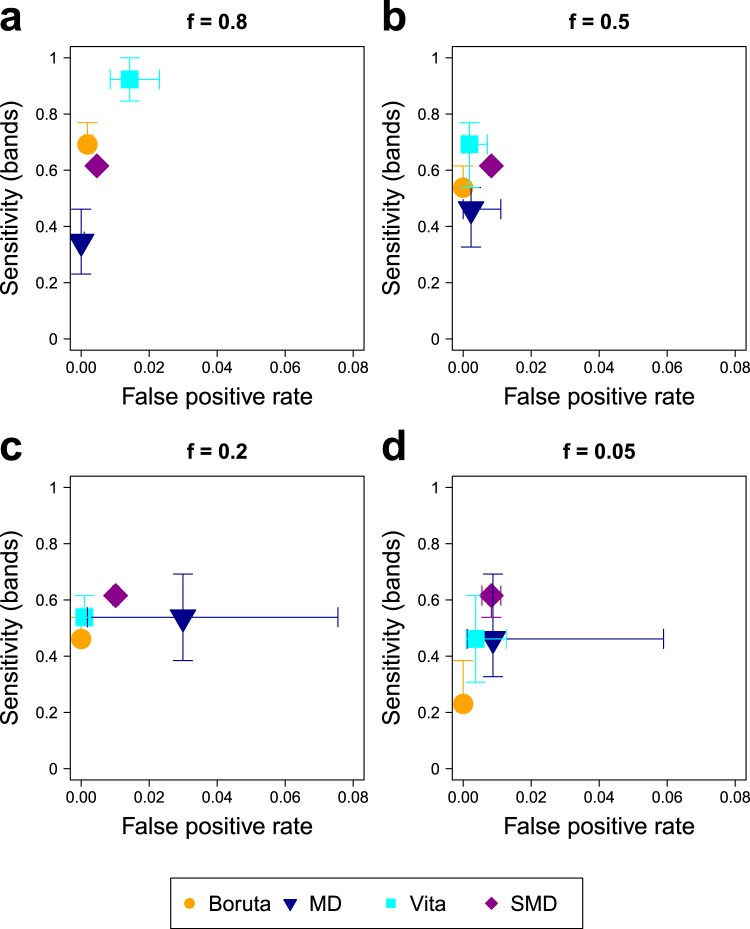
Performance comparison of the variable selection approaches on the SERS data sets with *f* = 0.8 (**a**), 0.5 (**b**), 0.2 (**c**), and 0.05 (**d**). Sensitivity of band identification and false positive rate are shown. Each subfigure displays the median over all 50 replicates of each method using different plotting symbols and colors. This figure was generated using the software R^33^ (version 3.5.2).

Figure 2 shows the results for the different SERS data sets and approaches. An optimal approach would be in the upper left corner of each of the plots. Vita shows the highest sensitivities (about 0.7 and almost 1) of all approaches for data sets with medium (*f* = 0.5) and high (*f* = 0.8) amounts of characteristic spectra, respectively. For the latter data set, however, this method also features a relatively high false positive rate of ca. 2%. The reason for this can be attributed to inaccurate assumptions of this method. Here it is assumed that a very high number of unimportant variables are present to obtain an accurate estimation of null variables by non-positive permutation importance scores. However, this assumption is flawed when a data set with high amounts of important variables is analyzed. The difficulties of this approach were also indicated by the developers^23^ expecting a poor performance if only few non-positive importance scores are observed. Probably the impact of this false assumption will be more serious in experimental SERS data since they are much more complex and, consequently will contain even less completely unimportant bands and variables.

In contrast, Boruta shows very few falsly detected variables indicated by a false positive rate of close to zero for all data sets. However this approach features the lowest sensitivity of about 0.4 to 0.5 and 0.2 to 0.4 for data sets with low (*f* = 0.2) and very low (*f* = 0.05) amounts of characteristic SERS spectra, respectively.

Minimal depth (MD) performs comparatively weak in this comparison study: For high (*f* = 0.8) and medium (*f* = 0.5) amounts of the characteristic spectra it shows the lowest sensitivities of around 0.4 and for low (*f* = 0.2) and very low (*f* = 0.05) amounts it shows very high false positive rates reaching nearly 8%. The reason for the latter probably is that in situations when few or no causal variables are present, variables from the bands of the background spectra are preferably chosen in the decision trees leading to selection of those variables by MD. This assumption is supported by the results of the null scenario that features high false positive rates for MD (Fig. S8) and that shows that individual variables are preferably falsly selected by MD (Fig. S7). This problem, however, probably is less expressed in more complex experimental data because a higher amount of non-important background signals is present here due to high numbers of different biomolecules in the samples.

The relatively low sensitivity of MD for the first two data sets (Fig. 2(a,b)) is probably caused by the fact that all of the variables containing similar information for the separation are in competition to be selected as primary split variable in the decision trees. Hence, only a small amount of the variables is needed in the trees for reliable separation and high numbers of variables, even though they contain the relevant information for classification, are not needed in the tree. This assumption is supported by the Figs. S3 and S4 because MD does not select any individual variable with a selection frequency of more than 70%. Experimental SERS data sets also contain co-occuring SERS signals that provide similar information for the classification. Nevertheless, this difficulty is expected to be less expressed here, as well, because this information is not static and completely interchangable like in the simulated data that is based on fixed single spectra. Instead, individual SERS signals in experimental data are influenced by the local environment and interactions of biomolecules with each other and the SERS nanoprobes. Hence, the causalities in experimental data are generally more complex than in this simulation and co-occuring SERS signals contain more different information.

Interestingly surrogate minimal depth (SMD) performs similarly for all four data sets. The sensitivity is at around 0.6, which is the second and third highest value for data sets with medium (*f* = 0.5) and high (*f* = 0.8) amounts of characteristic spectra, respectively, and the highest values for data sets with low (*f* = 0.2) and very low (*f* = 0.05) amounts. For this approach the false positive rate is also relatively constant at ca. 1%. The reason for this constant and very different performance to MD results from the incorporation of variable relations in the selection process. As a result, the similarity of the variables regarding their classification abilities enhances the abilities of the method to select important variables instead of reducing it. This is also why the sensitivities of SMD utilizing all important variables in comparison to the sensitivities of band identification show more similar values than the respective sensitivities of the other methods (compare Figs. 2 to S9). In the null scenario (Figs. S7d and S8) SMD selects the variable 679 cm^−1^ very frequently. Since this variable is never selected by MD, the reason for this must be that it shows high correlations to many other variables and, thus, is selected frequently as surrogate variable in the decision trees. This demonstrates the susceptibility of SMD to select variables exclusively because of their relations, which is also discussed in the original publication of this method^26^.

In conclusion, Boruta should be applied when the number of falsely detected variables should be minimized. SMD should be preferred when data is analyzed that is characterized by low amounts of characteristic information.

### Analysis of variable relations

**Figure 3:**
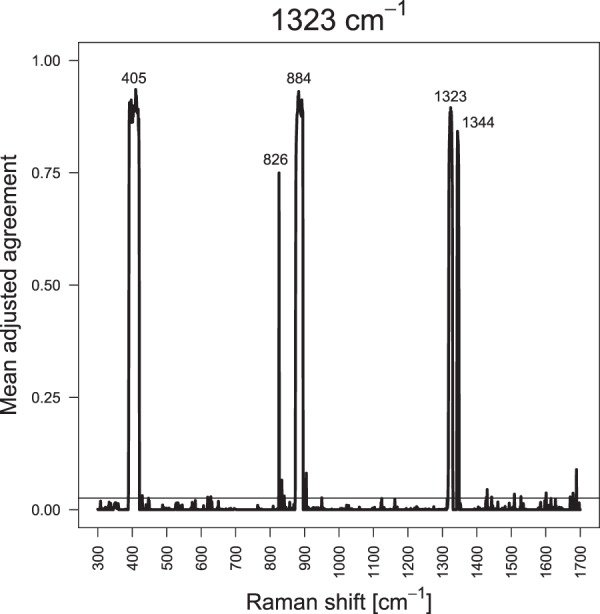
Mean adjusted agreement of the additional spectral variables for the variable at 1323 cm^−1^ using one replicate of the data set with f = 0.8. The threshold obtained with t = 1 (horizontal line) is applied to separate related variables with higher values from unrelated variables with lower values. This figure was generated using the software R^33^ (version 3.5.2).

The second characteristic of the SERS data sets that was analyzed was the relations of the variables. To find related variables SMD calculates the mean adjusted agreement and compares it to a threshold to select variables with higher values. Figure 3 exemplarily displays the mean adjusted agreement of all variables to find related variables for the band maximum at 1323 cm^−1^.

**Figure 4:**
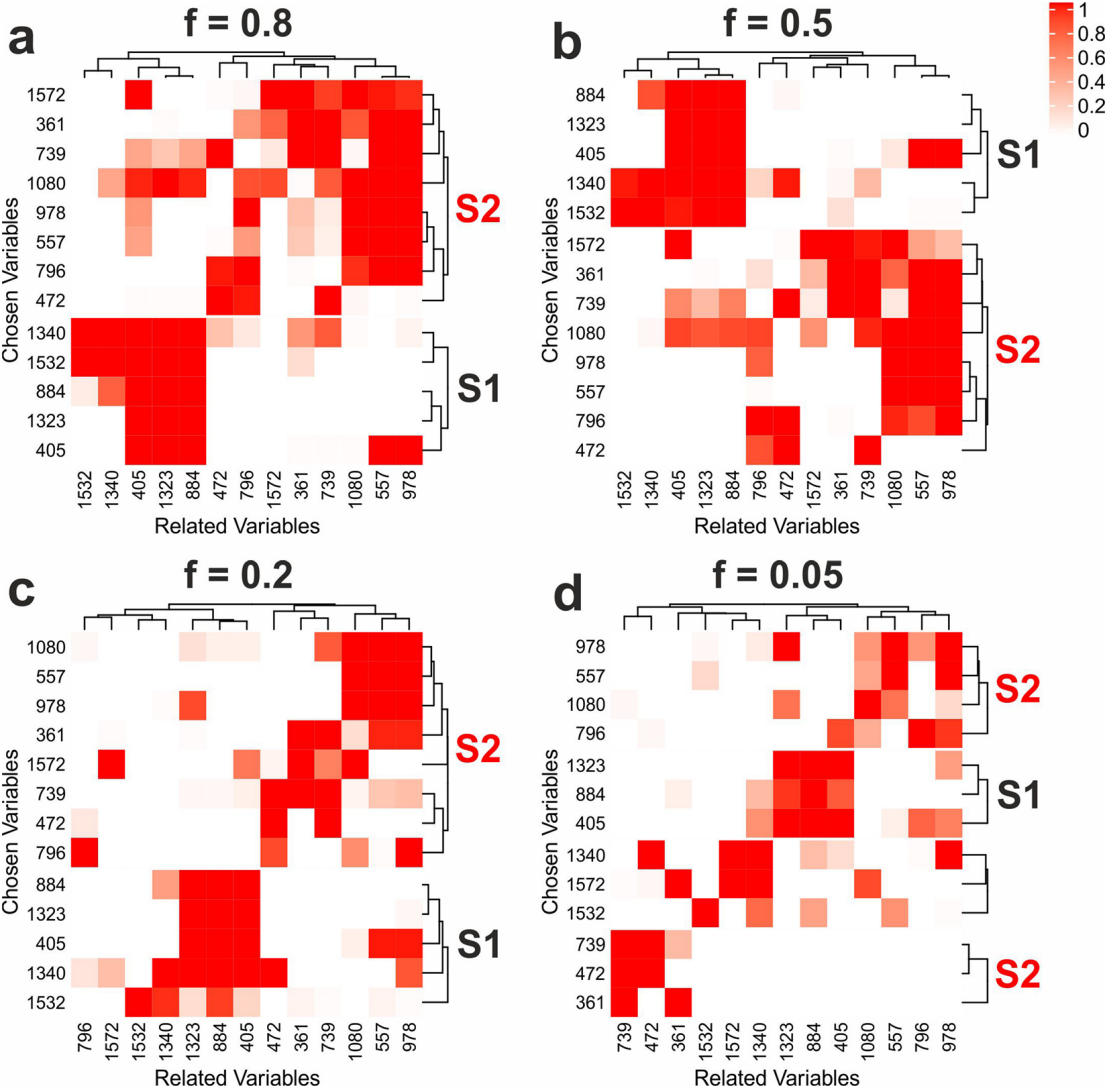
Results of variable relations analysis for the SERS data sets with *f* = 0.8 (**a**), 0.5 (**b**), 0.2 (**c**), and 0.05 (**d**). Heatmaps of the selection frequencies using all band maxima of the characteristic spectra as chosen and also as potentially related variables over all 50 replicates are shown. K-means clustering with Euclidean distance was applied and groups that are characteristic for a single spectrum are labeled with S1 (single spectrum 1) and S2 (single spectrum 2). This figure was generated using the software R^33^ (version 3.5.2) and CorelDRAW (version 19.1.0.419).

In order to accomplish a straightforward analysis of the relations of the characteristic bands, a detailed analysis was conducted for the 13 maxima band variables of the characteristic single spectra using only them as potentially related variables. Figure 4 shows heatmaps visualizing the proportion of replicates in which the respective relations were detected. The heatmaps are obviously not symmetric, meaning that the relation of variable *a* to variable *b* is not the same as the relation of variable *b* to variable *a*. This is reasonable, since the selection frequencies are based on the non-symmetric parameter mean adjusted agreement^26^. For the data sets with high, medium and low numbers of characteristic spectra (*f* = 0.8, 0.5, 0.2) the evaluated variables clearly classify in two groups that can be assigned to the two characteristic single spectra (labeled as S1 and S2). However, the analysis of the data set with *f* = 0.05 does not show this clear separation, but a large proportion of the variables build small groups that only consist of variables of the same characteristic spectrum. These characteristic groups are not present in the null scenario, that is characterized by three clusters with bands from both spectra (Fig. S10a). This confirms that the relations in Fig. 4 are caused by the characteristic information in the data sets. Interestingly, also in this scenario related variables are identified that can be assigned to co-occuring bands of the other single spectra. This is especially apparent when the maximum peak positions of the single spectra 8 and 12 are analyzed (Fig. S10b).

Since the single spectra represent different types of SERS information that individually co-occur in the SERS data sets it is obvious that this analysis is capable to distinguish between co-occuring SERS signals, especially when they are causal for the analyzed outcome. In a first application to experimental SERS data it has been demonstrated that this information is very useful to illuminate complex biological processes in cells like the interaction of antidepressants and lipids^35^.

### Evaluation of predefined spectral groups

One advantage of machine learning approaches for the analysis of spectroscopic data is the possibility to include previous knowledge. In the results presented so far, only the class information was included in the RF classification. However, the evaluation of variable selection techniques was adapted to include preknowledge about the SERS bands to analyze their selection frequency instead of single spectral variables for convenient comparison. In this paragraph the idea to include pre-knowledge is further developed by the application of so-called pathway-guided approaches that were developed to include external biological information into the analysis of omics data. These methods evaluate predefined variable groups instead of individual variables and select groups that are important for the outcome. A recent comparison study shows that self-sufficient methods that only use group variables and competing methods that compare the performance of group variables to variables outside of the group perform differently in high-dimensional data sets^32^. Hence, one from both of these types was used in the comparison on SERS data sets: Learner of Functional Enrichment (LeFE) was used as competing method and prediction error (PE) as self-sufficient method.

**Figure 5:**
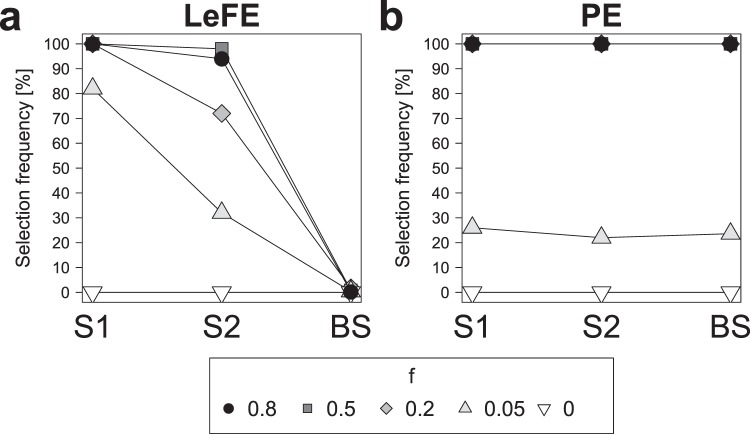
Performance comparison of the approaches Learner of Functional Enrichment (LeFE, **a**) and prediction error (PE, **b**). Selection frequencies over all 50 replicates for the SERS data sets with *f* = 0.8, 0.5, 0.2, 0.05, and 0 are shown. In the analysis the bands of the twelve single spectra were used as predefined groups and the performances of spectrum 1 (S1) and 2 (S2) are individually displayed while the performance of the background spectra 3 to 12 (BS) is averaged. This figure was generated using the software R^33^ (version 3.5.2).

Figure 5 displays the results for the analysis utilizing the bands of each of the twelve single spectra as a variable group. LeFE shows high selection frequencies for the characteristic groups ranging from 82% (S1) and 32% (S2) for data sets with very low amounts of characteristic spectra (*f* = 0.05) to over 90% for data sets with medium and high amounts (*f* = 0.5, 0.8) (Fig. 5a). Furthermore, all data sets show selection frequencies of nearly 0% for the groups that were built from the background spectra (BS).

PE shows selection frequencies of 100% for SERS data sets with *f* = 0.8, 0.5 and 0.2 and of 22 to 26% for *f* = 0.05 for the two characteristic groups S1 and S2 (Fig. 5b). However, PE obtains similar values for the background spectra 3 to 12 (BS). The reason for this probably is that the variables in the characteristic and non-characteristic groups overlap. Since PE features a very high empirical power^32^ this results in the selection of all predefined groups. Apparently self-sufficient methods are not suitable for the analysis of SERS data since overlapping variables will always be present here.

The comparison to other variables outside of the group as it is conducted in competing methods like LeFE is obviously necessary for reliable variable group evaluation of SERS data. Hence, LeFE could in future applications be utilized to evaluate SERS data sets for differences in specific SERS signals of biomolecule groups like proteins or lipids. In addition, the SERS signals of individual substances like drugs or biomolecules that are suspected to be relevant for the considered research question could be exploited to directly test for differences regarding those particular substances.

## Conclusions and Outlook

In conclusion, this study shows that the combination of random forest (RF) based approaches and surface-enhanced Raman scattering (SERS) is very promising to analyze complex biological samples. To be more precise, RF approaches expand the possibilities of SERS data analysis to obtain accurate and sophisticated interpretation of complex SERS data and enable the incorporation of previous knowledge about the SERS signals into the analysis. Regarding the selection of important variables: Boruta should be applied for a minimum number of falsly detected variables and surrogate minimal depth (SMD) when the data contains few characteristic information. SMD can additionally be applied to analyze variable relations and to separate different co-occuring SERS signals. In order to include knowledge about spectral groups it is shown that the competing method Learner of Functional Enrichment (LeFE) should be applied instead of the self-sufficient approach prediction error (PE).

In the future, it is planned to apply and validate the obtained results on experimental data and to expand the application of RF methods to analyze further characteristics of SERS data. As an example, peak shifts could be analyzed to derive environmental and structural changes of biomolecules. In this context, laboratory experiments will be combined with simulations based on the simulation framework presented here to obtain a comprehensive view on the complex properties of SERS experiments.

## Supplementary information

Supplementary Information 1.

Supplementary Information 2.

## Data Availability

An R markdown script to generate the SERS data is provided as supplementary material.
