# Supplementary material for: Application of random forest based approaches to surface-enhanced Raman scattering data
Source: Sci Rep. 2020 Mar 25;10:5436. doi: 10.1038/s41598-020-62338-8 (PMC7096517; doi:10.1038/s41598-020-62338-8)
Supplement: Supplementary file 1 — Supplementary Information 1. [file 41598_2020_62338_MOESM1_ESM.zip › execute_simulation.html]

# Single spectra simulation

```
# define folder locations and load packages and functions
require(R.utils)          ## loadObject(), saveObject()
require(metricsgraphics)  ## interactive graphs

current.dir = getwd()
data.dir.sim = file.path(current.dir, "data")
source(paste0(current.dir,"/functions.R"))


dir.create(data.dir.sim)


rerun = FALSE

rep = 50
total.single.spectra = 12
no.bands = c(3,10)
baseline = 200
noise = 50
width.bands = c(5,20)
range.wavelength = c(300,1700)
res = 1
int.width = c(1000,10000)

set.seed(4321)
single_spectra = simulate_spectra(no.spectra = total.single.spectra, 
                                  no.bands = no.bands, 
                                  baseline = baseline, 
                                  noise = noise, 
                                  width.bands = width.bands, 
                                  range.wavelength = range.wavelength, 
                                  res = res, 
                                  int.width = int.width)
    saveObject(single_spectra,file = paste0(data.dir.sim,"/single_spectra.RData"))
    make.infopw(single_spectra = single_spectra,
                       data.dir.sim = data.dir.sim)
```

- 12 single spectra (wavelength from 300 to 1700 with resolution 1) were simulated:
  - number of bands range from 3 to 10
  - width of bands range from 5 to 20
  - intensity ranges from 1000 to 10^{4}
  - baseline is set to 200
  - noise is set to 50

```
i = 1
plot.data = as.data.frame(cbind(c(300:1700),as.numeric(single_spectra$spectra[,i])))
colnames(plot.data) = c("wavelength","y")
mjs_plot(plot.data, x = wavelength, y = y, title = paste0("Spectrum ", i)) %>%
  mjs_labs(x = "Raman shift [cm-1]", y = "Raman intensity")
```

```
i = 2
plot.data = as.data.frame(cbind(c(300:1700),as.numeric(single_spectra$spectra[,i])))
colnames(plot.data) = c("wavelength","y")
mjs_plot(plot.data, x = wavelength, y = y, title = paste0("Spectrum ", i)) %>%
  mjs_labs(x = "Raman shift [cm-1]", y = "Raman intensity")
```

```
i = 3
plot.data = as.data.frame(cbind(c(300:1700),as.numeric(single_spectra$spectra[,i])))
colnames(plot.data) = c("wavelength","y")
mjs_plot(plot.data, x = wavelength, y = y, title = paste0("Spectrum ", i)) %>%
  mjs_labs(x = "Raman shift [cm-1]", y = "Raman intensity")
```

```
i = 4
plot.data = as.data.frame(cbind(c(300:1700),as.numeric(single_spectra$spectra[,i])))
colnames(plot.data) = c("wavelength","y")
mjs_plot(plot.data, x = wavelength, y = y, title = paste0("Spectrum ", i)) %>%
  mjs_labs(x = "Raman shift [cm-1]", y = "Raman intensity")
```

```
i = 5
plot.data = as.data.frame(cbind(c(300:1700),as.numeric(single_spectra$spectra[,i])))
colnames(plot.data) = c("wavelength","y")
mjs_plot(plot.data, x = wavelength, y = y, title = paste0("Spectrum ", i)) %>%
  mjs_labs(x = "Raman shift [cm-1]", y = "Raman intensity")
```

```
i = 6
plot.data = as.data.frame(cbind(c(300:1700),as.numeric(single_spectra$spectra[,i])))
colnames(plot.data) = c("wavelength","y")
mjs_plot(plot.data, x = wavelength, y = y, title = paste0("Spectrum ", i)) %>%
  mjs_labs(x = "Raman shift [cm-1]", y = "Raman intensity")
```

```
i = 7
plot.data = as.data.frame(cbind(c(300:1700),as.numeric(single_spectra$spectra[,i])))
colnames(plot.data) = c("wavelength","y")
mjs_plot(plot.data, x = wavelength, y = y, title = paste0("Spectrum ", i)) %>%
  mjs_labs(x = "Raman shift [cm-1]", y = "Raman intensity")
```

```
i = 8
plot.data = as.data.frame(cbind(c(300:1700),as.numeric(single_spectra$spectra[,i])))
colnames(plot.data) = c("wavelength","y")
mjs_plot(plot.data, x = wavelength, y = y, title = paste0("Spectrum ", i)) %>%
  mjs_labs(x = "Raman shift [cm-1]", y = "Raman intensity")
```

```
i = 9
plot.data = as.data.frame(cbind(c(300:1700),as.numeric(single_spectra$spectra[,i])))
colnames(plot.data) = c("wavelength","y")
mjs_plot(plot.data, x = wavelength, y = y, title = paste0("Spectrum ", i)) %>%
  mjs_labs(x = "Raman shift [cm-1]", y = "Raman intensity")
```

```
i = 10
plot.data = as.data.frame(cbind(c(300:1700),as.numeric(single_spectra$spectra[,i])))
colnames(plot.data) = c("wavelength","y")
mjs_plot(plot.data, x = wavelength, y = y, title = paste0("Spectrum ", i)) %>%
  mjs_labs(x = "Raman shift [cm-1]", y = "Raman intensity")
```

```
i = 11
plot.data = as.data.frame(cbind(c(300:1700),as.numeric(single_spectra$spectra[,i])))
colnames(plot.data) = c("wavelength","y")
mjs_plot(plot.data, x = wavelength, y = y, title = paste0("Spectrum ", i)) %>%
  mjs_labs(x = "Raman shift [cm-1]", y = "Raman intensity")
```

```
i = 12
plot.data = as.data.frame(cbind(c(300:1700),as.numeric(single_spectra$spectra[,i])))
colnames(plot.data) = c("wavelength","y")
mjs_plot(plot.data, x = wavelength, y = y, title = paste0("Spectrum ", i)) %>%
  mjs_labs(x = "Raman shift [cm-1]", y = "Raman intensity")
```

# SERS spectra simulation

- Spectrum 1 and spectrum 2 were used as specific spectra for the two groups (datasets)
- Spectrum 3, 4, 5, 6, 7, 8, 9, 10, 11, and 12 were used to build the background
- Data sets with 2\*500 SERS spectra were created using 2 to 5 single spectra for each SERS spectrum
- The percentage of the characterstic spectrum in the SERS spectrum ranged from 0.1 to 0.8 (this parameter is called w in the paper)
- The percentage of the respective background spectrum in the SERS background ranged from 0.1 to 0.8
- 5 different data sets with different values for the variable perc.char (0.8, 0.5, 0.2, 0.05, 0) were generated. This variable defines which percentage of the SERS spectra contain the characteristic spectrum and is called f in the paper.

```
perc.char = 0.8 # percentage of the SERS spectra that contain the characteristic spectrum
name = paste0("Data1_perc.char_",perc.char)

data.sub.dir = file.path(data.dir.sim, name)
dir.create(data.sub.dir)
if (rerun) {
  for (i in 1:rep) {
    set.seed(42 + (i*815*perc.char)) 
    spectra.with1 = combine_spectra(no.spectra = no.spectra,
                                    spectrum_char = single_spectra$spectra[,1],
                                    spectra_back = single_spectra$spectra[,c(-1,-2)],
                                    perc.char = perc.char,
                                    range.num.single.spec = range.num.single.spec,
                                    perc.char.range = perc.char.range, 
                                    perc.back.range = perc.back.range,
                                    name.DS = "DS1", 
                                    vectornorm = TRUE)
    saveObject(spectra.with1,file = paste0(data.sub.dir,"/DS1_",i,".RData"))
    
    spectra.with2 = combine_spectra(no.spectra = no.spectra,
                                    spectrum_char = single_spectra$spectra[,2],
                                    spectra_back = single_spectra$spectra[,c(-1,-2)],
                                    perc.char = perc.char,
                                    range.num.single.spec = range.num.single.spec,
                                    perc.char.range = perc.char.range, 
                                    perc.back.range = perc.back.range,
                                    name.DS = "DS1", 
                                    vectornorm = TRUE)
    saveObject(spectra.with2,file = paste0(data.sub.dir,"/DS2_",i,".RData"))
  }
}
```

```
perc.char = 0.5 # percentage of the SERS spectra that contain the characteristic spectrum
name = paste0("Data1_perc.char_",perc.char)

data.sub.dir = file.path(data.dir.sim, name)
dir.create(data.sub.dir)
if (rerun) {
  for (i in 1:rep) {
    set.seed(42 + (i*815*perc.char)) 
    spectra.with1 = combine_spectra(no.spectra = no.spectra,
                                    spectrum_char = single_spectra$spectra[,1],
                                    spectra_back = single_spectra$spectra[,c(-1,-2)],
                                    perc.char = perc.char,
                                    range.num.single.spec = range.num.single.spec,
                                    perc.char.range = perc.char.range, 
                                    perc.back.range = perc.back.range,
                                    name.DS = "DS1", 
                                    vectornorm = TRUE)
    saveObject(spectra.with1,file = paste0(data.sub.dir,"/DS1_",i,".RData"))
    
    spectra.with2 = combine_spectra(no.spectra = no.spectra,
                                    spectrum_char = single_spectra$spectra[,2],
                                    spectra_back = single_spectra$spectra[,c(-1,-2)],
                                    perc.char = perc.char,
                                    range.num.single.spec = range.num.single.spec,
                                    perc.char.range = perc.char.range, 
                                    perc.back.range = perc.back.range,
                                    name.DS = "DS1", 
                                    vectornorm = TRUE)
    saveObject(spectra.with2,file = paste0(data.sub.dir,"/DS2_",i,".RData"))
  }
}
```

```
perc.char = 0.2 # percentage of the SERS spectra that contain the characteristic spectrum
name = paste0("Data1_perc.char_",perc.char)

data.sub.dir = file.path(data.dir.sim, name)
dir.create(data.sub.dir)
if (rerun) {
  for (i in 1:rep) {
    set.seed(42 + (i*815*perc.char)) 
    spectra.with1 = combine_spectra(no.spectra = no.spectra,
                                    spectrum_char = single_spectra$spectra[,1],
                                    spectra_back = single_spectra$spectra[,c(-1,-2)],
                                    perc.char = perc.char,
                                    range.num.single.spec = range.num.single.spec,
                                    perc.char.range = perc.char.range, 
                                    perc.back.range = perc.back.range,
                                    name.DS = "DS1", 
                                    vectornorm = TRUE)
    saveObject(spectra.with1,file = paste0(data.sub.dir,"/DS1_",i,".RData"))
    
    spectra.with2 = combine_spectra(no.spectra = no.spectra,
                                    spectrum_char = single_spectra$spectra[,2],
                                    spectra_back = single_spectra$spectra[,c(-1,-2)],
                                    perc.char = perc.char,
                                    range.num.single.spec = range.num.single.spec,
                                    perc.char.range = perc.char.range, 
                                    perc.back.range = perc.back.range,
                                    name.DS = "DS1", 
                                    vectornorm = TRUE)
    saveObject(spectra.with2,file = paste0(data.sub.dir,"/DS2_",i,".RData"))
  }
}
```

```
perc.char = 0.05 # percentage of the SERS spectra that contain the characteristic spectrum
name = paste0("Data1_perc.char_",perc.char)

data.sub.dir = file.path(data.dir.sim, name)
dir.create(data.sub.dir)
if (rerun) {
  for (i in 1:rep) {
    set.seed(42 + (i*815*perc.char)) 
    spectra.with1 = combine_spectra(no.spectra = no.spectra,
                                    spectrum_char = single_spectra$spectra[,1],
                                    spectra_back = single_spectra$spectra[,c(-1,-2)],
                                    perc.char = perc.char,
                                    range.num.single.spec = range.num.single.spec,
                                    perc.char.range = perc.char.range, 
                                    perc.back.range = perc.back.range,
                                    name.DS = "DS1", 
                                    vectornorm = TRUE)
    saveObject(spectra.with1,file = paste0(data.sub.dir,"/DS1_",i,".RData"))
    
    spectra.with2 = combine_spectra(no.spectra = no.spectra,
                                    spectrum_char = single_spectra$spectra[,2],
                                    spectra_back = single_spectra$spectra[,c(-1,-2)],
                                    perc.char = perc.char,
                                    range.num.single.spec = range.num.single.spec,
                                    perc.char.range = perc.char.range, 
                                    perc.back.range = perc.back.range,
                                    name.DS = "DS1", 
                                    vectornorm = TRUE)
    saveObject(spectra.with2,file = paste0(data.sub.dir,"/DS2_",i,".RData"))
  }
}
```

```
perc.char = 0 # percentage of the SERS spectra that contain the characteristic spectrum
name = paste0("Data1_perc.char_",perc.char)

data.sub.dir = file.path(data.dir.sim, name)
dir.create(data.sub.dir)
if (rerun) {
  for (i in 1:rep) {
    set.seed(42 + (i*815*perc.char)) 
    spectra.with1 = combine_spectra(no.spectra = no.spectra,
                                    spectrum_char = single_spectra$spectra[,1],
                                    spectra_back = single_spectra$spectra[,c(-1,-2)],
                                    perc.char = perc.char,
                                    range.num.single.spec = range.num.single.spec,
                                    perc.char.range = perc.char.range, 
                                    perc.back.range = perc.back.range,
                                    name.DS = "DS1", 
                                    vectornorm = TRUE)
    saveObject(spectra.with1,file = paste0(data.sub.dir,"/DS1_",i,".RData"))
    
    spectra.with2 = combine_spectra(no.spectra = no.spectra,
                                    spectrum_char = single_spectra$spectra[,2],
                                    spectra_back = single_spectra$spectra[,c(-1,-2)],
                                    perc.char = perc.char,
                                    range.num.single.spec = range.num.single.spec,
                                    perc.char.range = perc.char.range, 
                                    perc.back.range = perc.back.range,
                                    name.DS = "DS1", 
                                    vectornorm = TRUE)
    saveObject(spectra.with2,file = paste0(data.sub.dir,"/DS2_",i,".RData"))
  }
}
```
