# Supplementary material for: Application of random forest based approaches to surface-enhanced Raman scattering data
Source: Sci Rep. 2020 Mar 25;10:5436. doi: 10.1038/s41598-020-62338-8 (PMC7096517; doi:10.1038/s41598-020-62338-8)
Supplement: Supplementary file 2 — Supplementary Information 2. [file 41598_2020_62338_MOESM2_ESM.pdf]

Supplementary information
“Application of random forest based approaches to surface-enhanced Raman scattering data”

Stephan Seifert

Contents

1 Supplementary figures **2**

2 References **12**

1 Supplementary figures

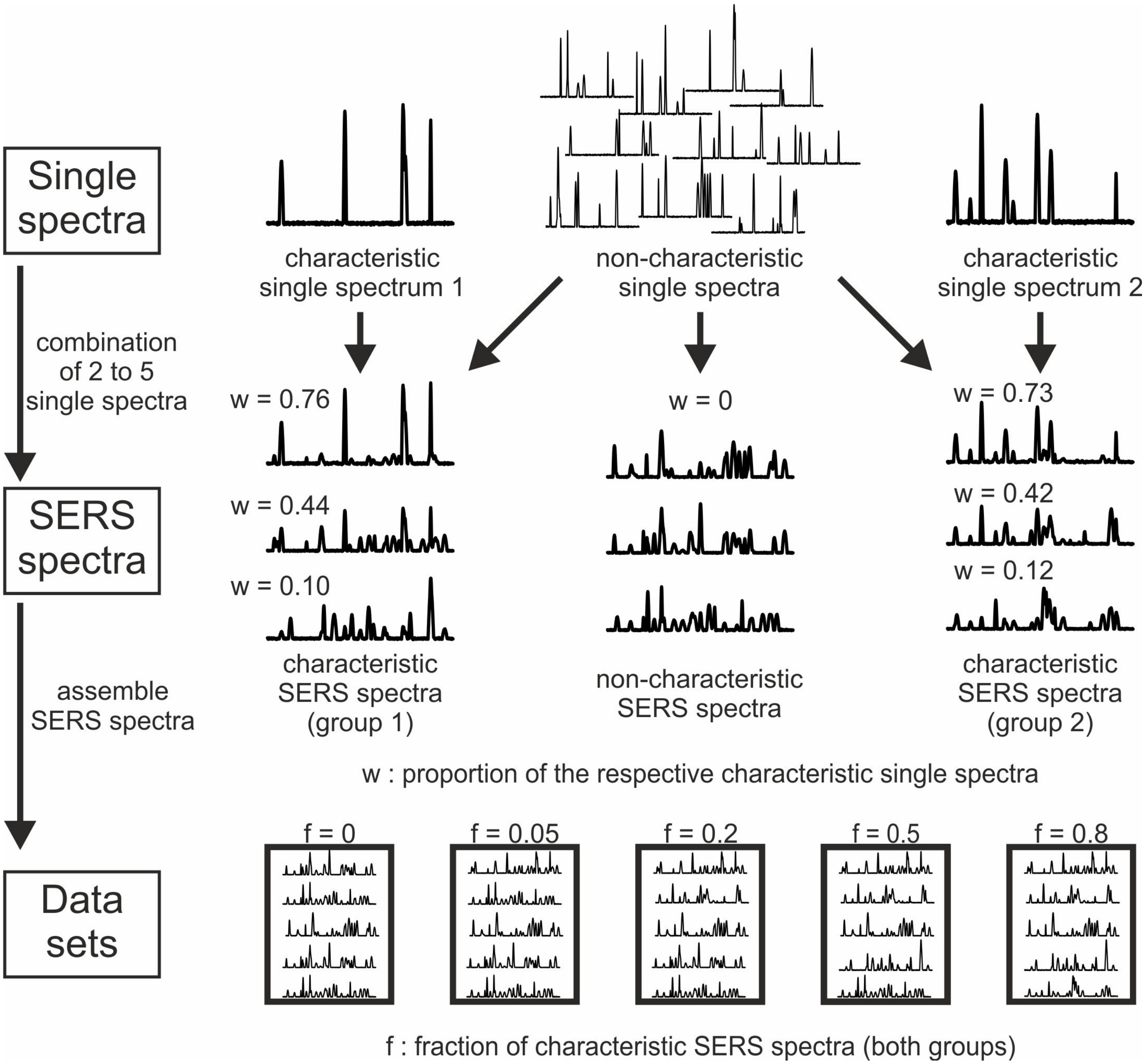

Figure S 1: Schematic diagram of the simulation of SERS data. This figure was generated using the software CorelDRAW (version 19.1.0.419).

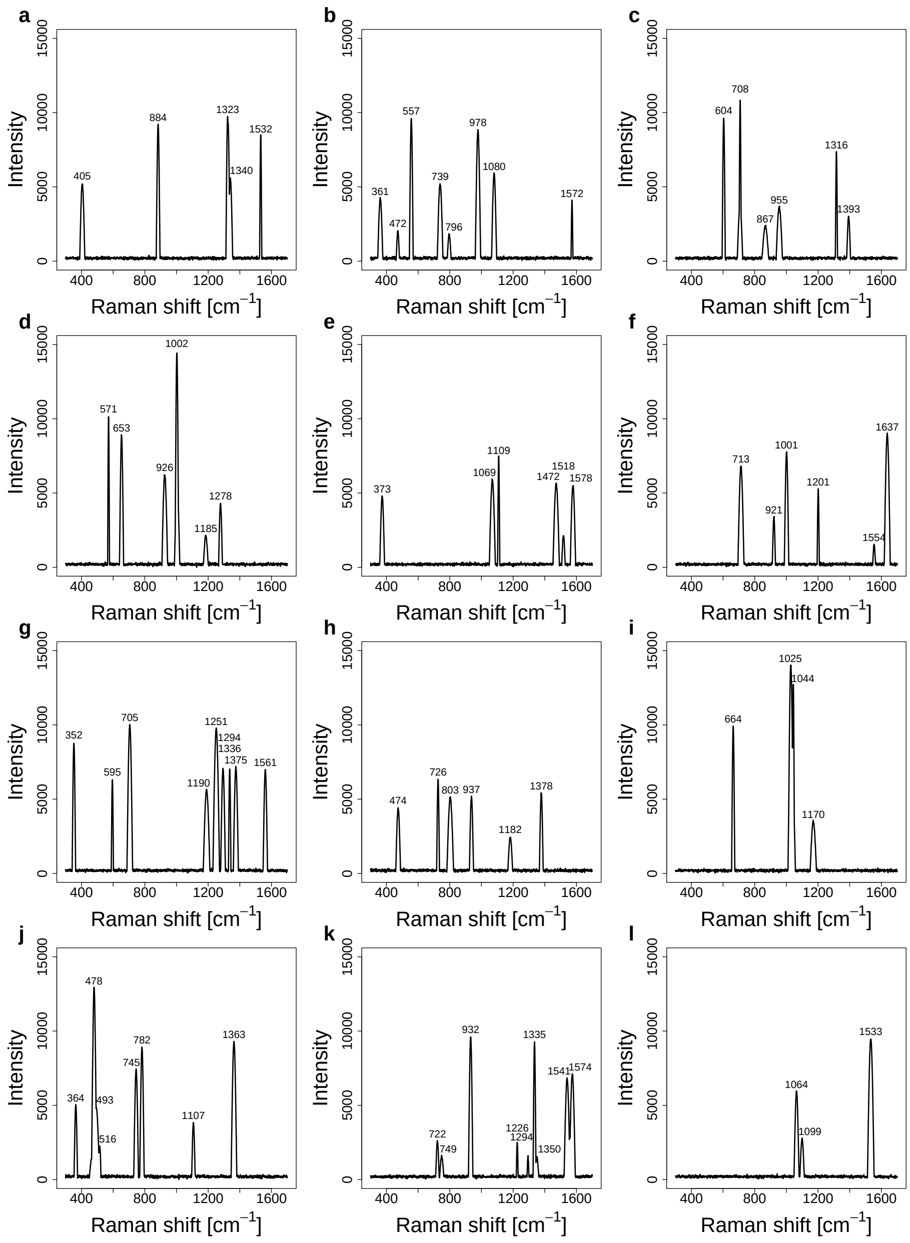

Figure S 2: The twelve single spectra utilized as characteristic (a+b) and background (c-l) SERS signals for the generation of SERS data sets. This figure was generated using the software R[1] (version 3.5.2).

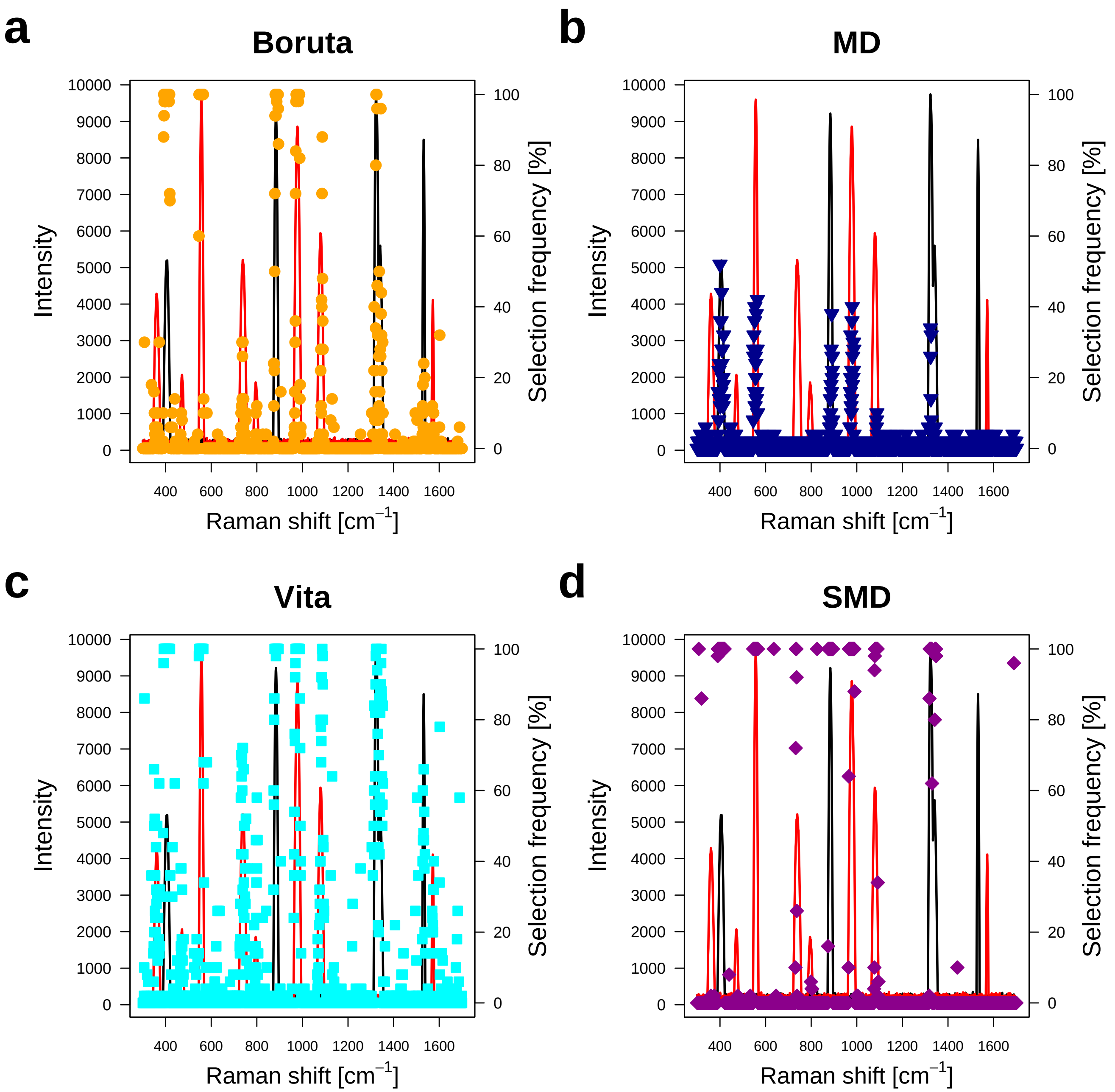

Figure S 3: Performance comparison of the variable selection approaches Boruta (a), minimal depth(MD, b), Vita (c), and surrogate minimal depth(SMD, d) on the SERS data set with f = 0.8. The characteristic single spectra are depicted in red and black, and the selection frequencies of each spectral variable over all 50 replicates is shown. This figure was generated using the software R[1] (version 3.5.2).

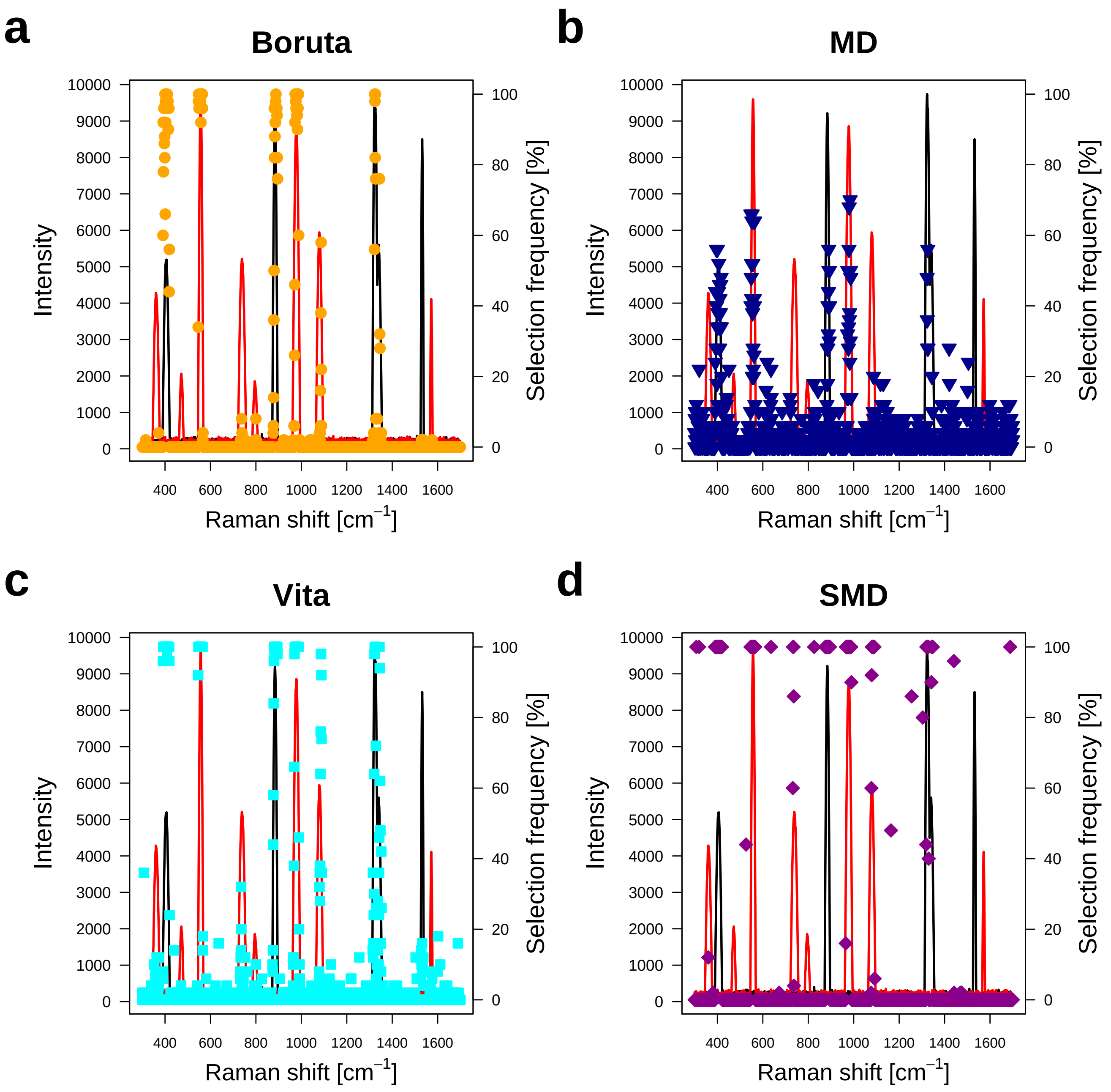

Figure S 4: Performance comparison of the variable selection approaches Boruta (a), minimal depth(MD, b), Vita (c), and surrogate minimal depth(SMD, d) on the SERS data set with f = 0.5. The characteristic single spectra are depicted in red and black, and the selection frequencies of each spectral variable over all 50 replicates is shown. This figure was generated using the software R[1] (version 3.5.2).

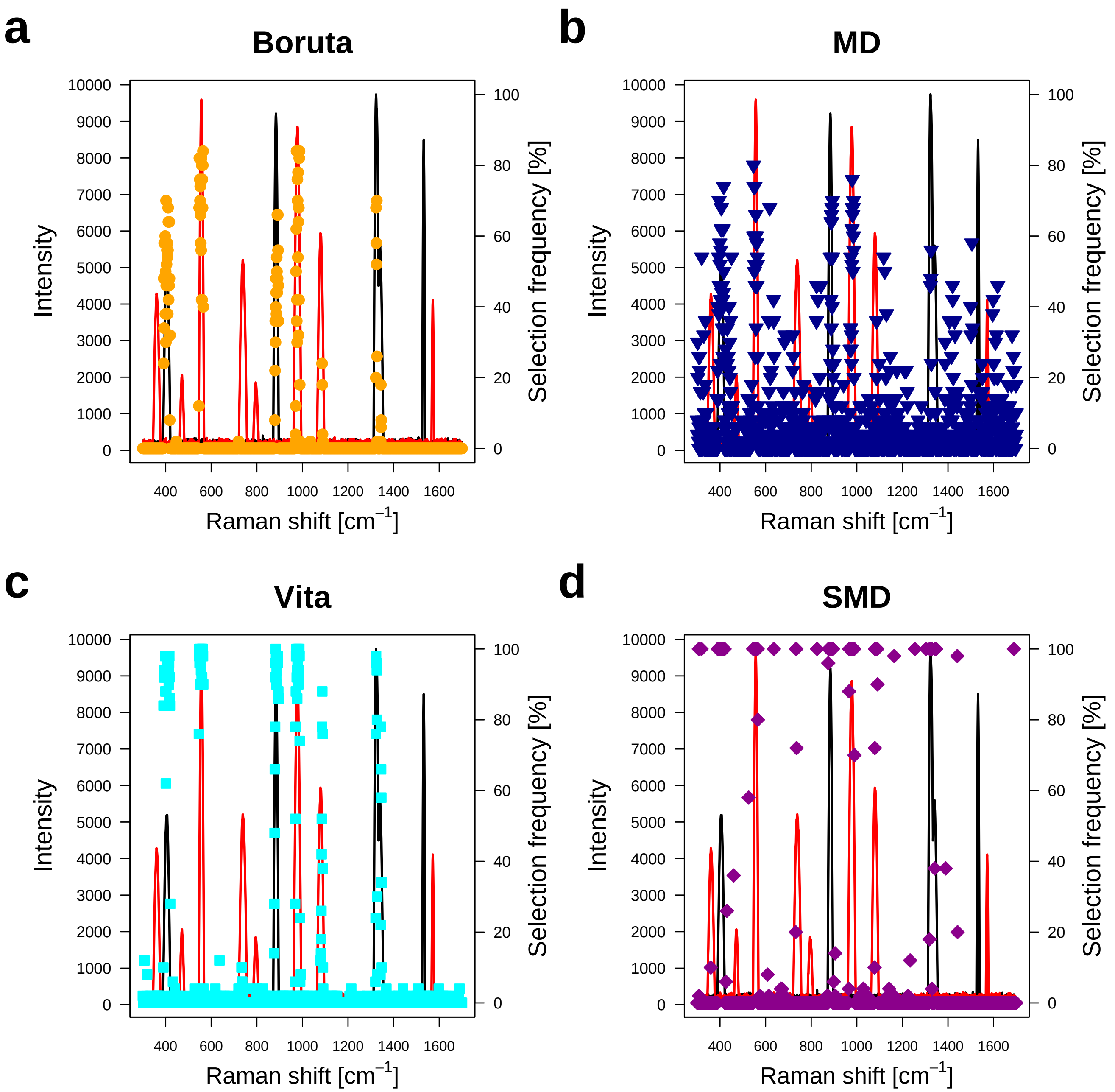

Figure S 5: Performance comparison of the variable selection approaches Boruta (a), minimal depth(MD, b), Vita (c), and surrogate minimal depth(SMD, d) on the SERS data set with f = 0.2. The characteristic single spectra are depicted in red and black, and the selection frequencies of each spectral variable over all 50 replicates is shown. This figure was generated using the software R[1] (version 3.5.2).

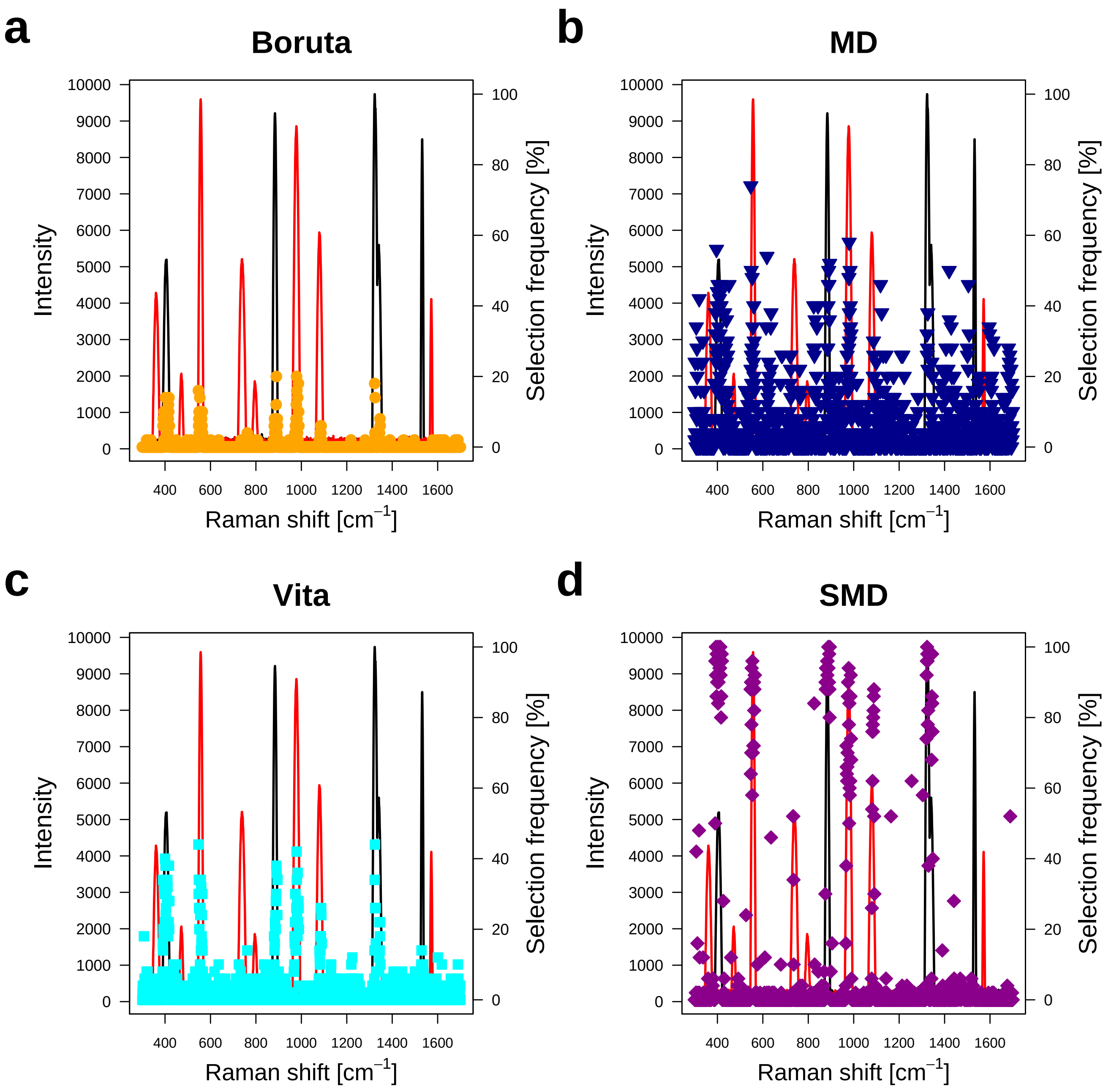

Figure S 6: Performance comparison of the variable selection approaches Boruta (a), minimal depth(MD, b), Vita (c), and surrogate minimal depth(SMD, d) on the SERS data set with f = 0.05. The characteristic single spectra are depicted in red and black, and the selection frequencies of each spectral variable over all 50 replicates is shown. This figure was generated using the software R[1] (version 3.5.2).

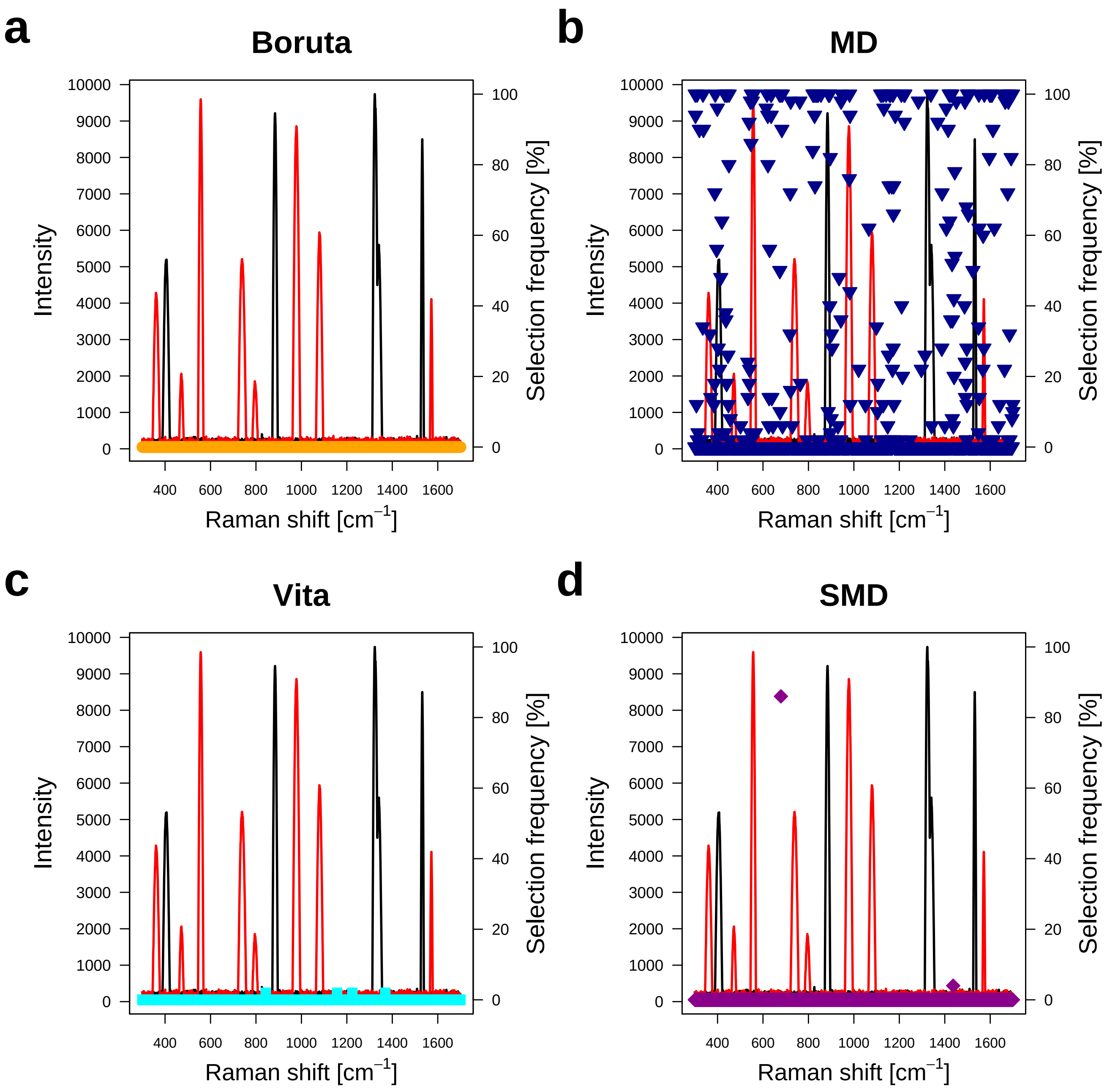

Figure S 7: Performance comparison of the variable selection approaches Boruta (a), minimal depth (MD, b), Vita (c), and surrogate minimal depth (SMD, d) on the SERS data set with f = 0. The characteristic single spectra are depicted in red and black, and the selection frequencies of each spectral variable over all 50 replicates is shown. This figure was generated using the software R[1] (version 3.5.2).

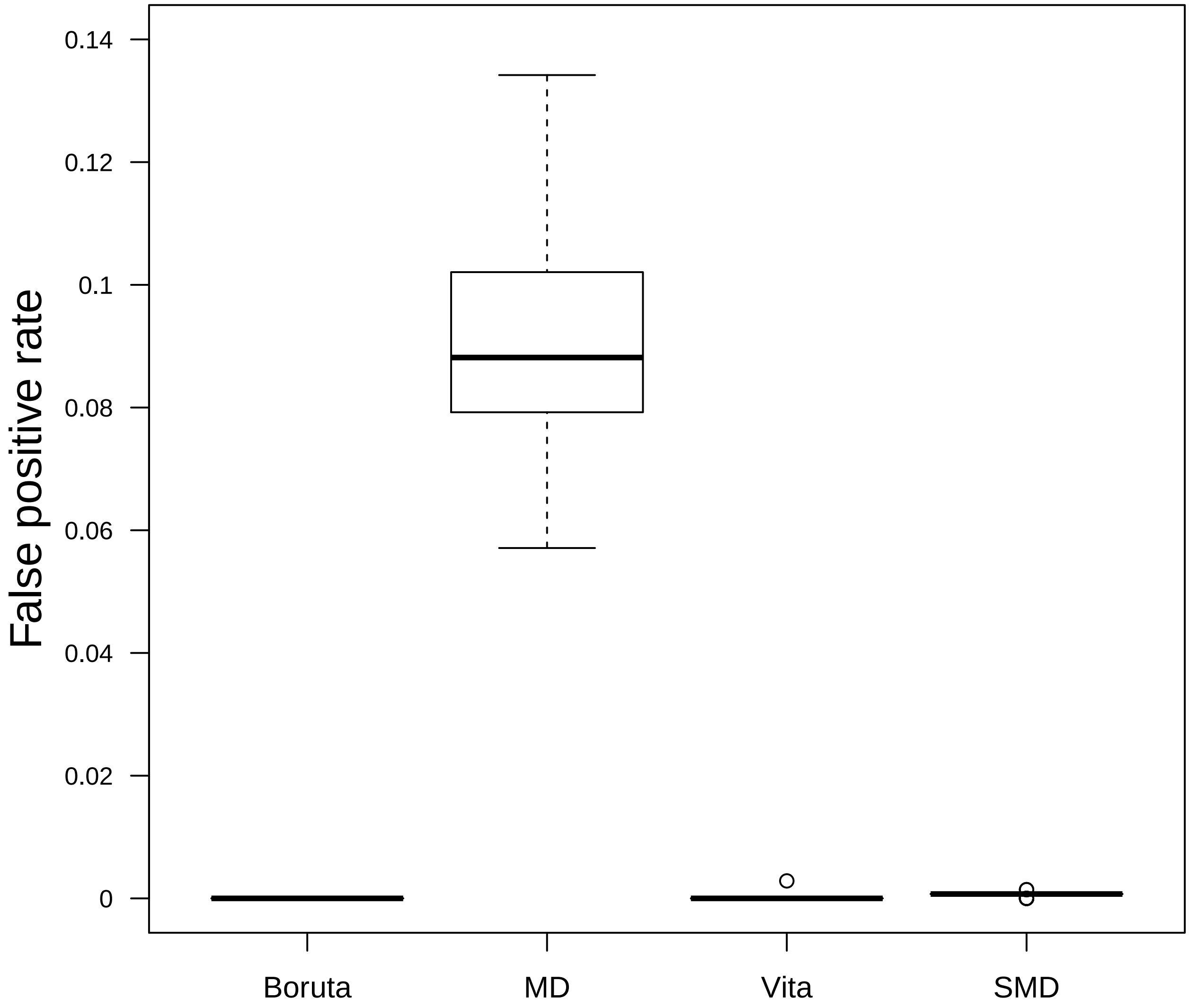

Figure S 8: Boxplots for the false positive rate of the variable selection approaches Boruta, minimal depth (MD), Vita, and surrogate minimal depth (SMD) applied on the SERS data set with f = 0 (null scenario). False positive rate was determined using the selection results for all 1401 spectral variables over 50 replicates. This figure was generated using the software R[1] (version 3.5.2).

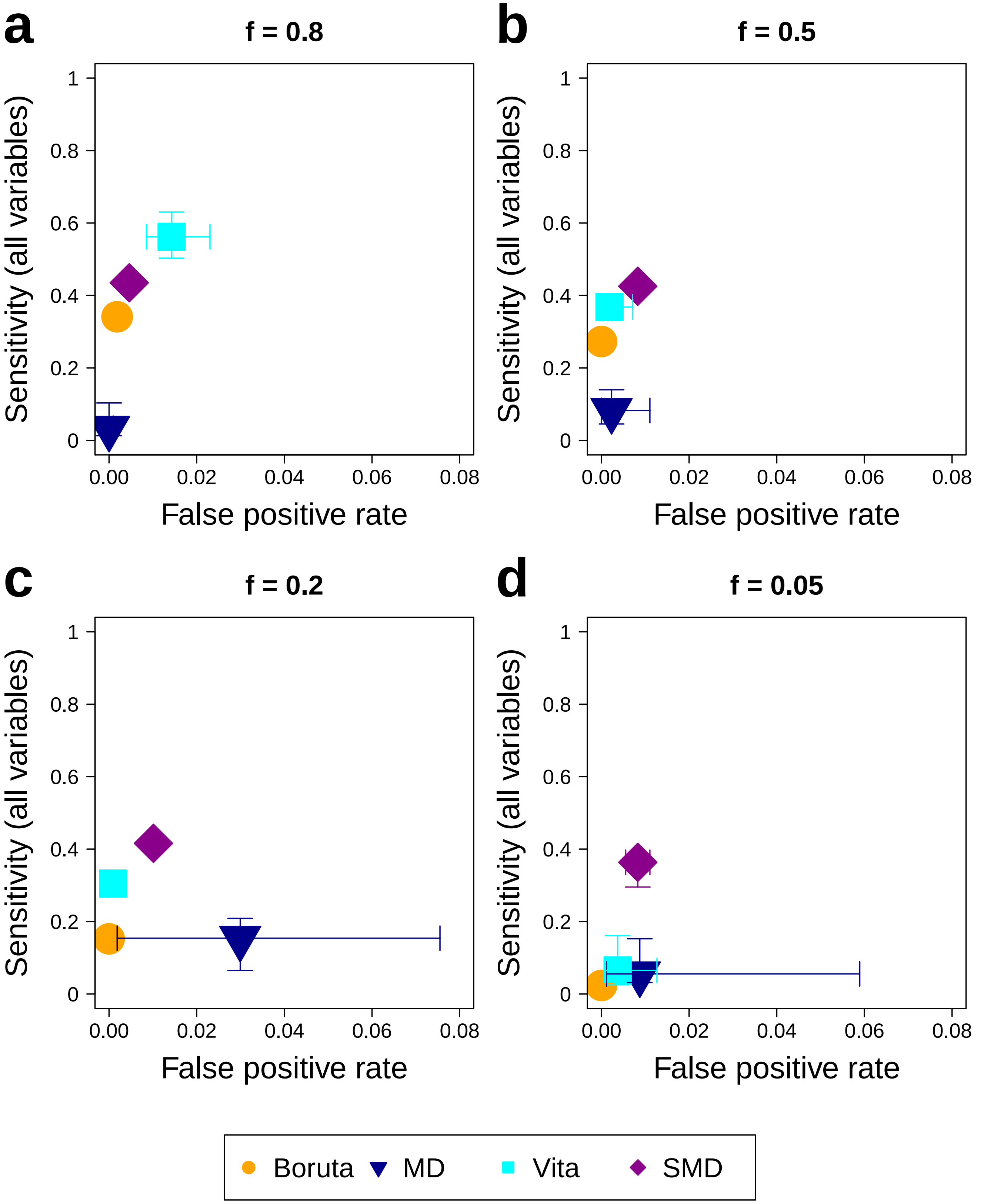

Figure S 9: Performance comparison of the variable selection approaches on the SERS data sets with f = 0.8 (a), 0.5 (b), 0.2 (c), and 0.05 (d). Sensitivity for all variables of the bands in the characteristic single spectra and false positive rate are shown. Each subfigure displays the median over all 50 replicates of each method using different plotting symbols and colors. This figure was generated using the software R[1] (version 3.5.2).

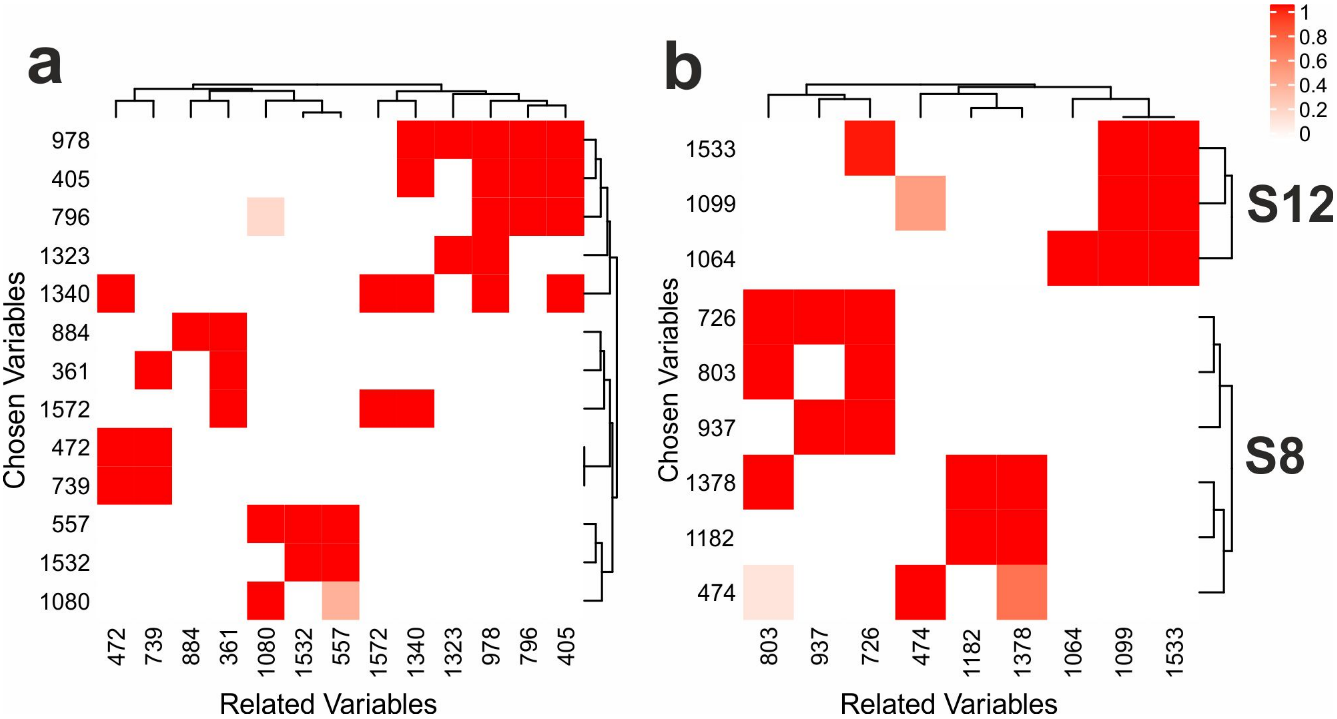

Figure S 10: Results of variable relations analysis for the SERS data sets with f = 0. Heatmaps of the selection frequencies using all band maxima of the characteristic spectra (a) and of the single spectra 8 and 12 (b) as chosen and also as potentially related variable over all 50 replicates are shown. K-means clustering with Euclidean distance was applied and in Figure b groups that are characteristic for a single spectrum are labeled with S8 (single spectrum 8) and S12 (single spectrum 12). This figure was generated using the software R[1] (version 3.5.2) and CorelDRAW (version 19.1.0.419).

2 References

[1] R Core Team (2013). R: A language and environment for statistical computing. R Foundation for Statistical Computing, Vienna, Austria. URL http://www.R-project.org/.
